# Molecular markers and cytogenetics to characterize a wheat-*Dasypyrum villosum* 3V (3D) substitution line conferring resistance to stripe rust

**DOI:** 10.1371/journal.pone.0202033

**Published:** 2018-08-29

**Authors:** Jie Zhang, Yun Jiang, Ying Wang, Yuanlin Guo, Hai Long, Guangbing Deng, Qian Chen, Pu Xuan

**Affiliations:** 1 Institute of Biotechnology and Nuclear Technology Research, Sichuan Academy of Agricultural Sciences, Chengdu, Sichuan, China; 2 Key Laboratory of Wheat Biology and Genetic Improvement on Southwestern China (Ministry of Agriculture), Chengdu, Sichuan, China; 3 Chengdu Institute of Biology, Chinese Academy of Sciences, Chengdu, Sichuan, China; 4 Institute of Agro-products Processing Science and Technology, Sichuan Academy of Agricultural Sciences, Chengdu, Sichuan, China; Institute of Genetics and Developmental Biology Chinese Academy of Sciences, CHINA

## Abstract

*Dasypyrum villosum* has been used as a valuable gene resource for disease resistances, yield increase and quality improvement in wheat. A novel wheat-*D*. *villosum* alien introgression line CD-3 was generated through hybridization between the common wheat Chinese Spring (CS) and a CS- *D*. *villosum* 3V addition line having considerably high stripe rust resistance, which enable the characterization of a potential new stripe rust resistance gene (s) derived from *D*. *villosum*. The results of non-denaturing fluorescent in situ hybridization (ND-FISH) showed that CD-3 contained 42 chromosomes, including a 3V chromosome pair, and the absence of both of the 3D chromosomes. PCR-based Landmark Unique Gene (PLUG) molecular marker analysis supported results from the FISH analysis, revealing CD-3 was a wheat-*D*. *villosum* 3V (3D) disomic substitution line. Resistant test of stripe rust on 52 plants of F_2_ generation (CD-3/CS), CD-3, CS and *D*.*villosum* have been conducted at seedling stage. 7 plants of F_2_ generation possessing two 3V chromosomes exhibited high resistance to stripe rust as CD-3 and *D*.*villosum*, 10 plants carrying one 3V chromosome and 35 plants without 3V chromosome were susceptive to stripe rust as CS. The result implied the high stripe rust resistance of CD-3 should be controlled by recessive gene(s) originating from *D*.*villosum*. To rapidly detect chromosome 3V in the genetic background of wheat, we developed a novel Sequence Characterized Amplified Region (SCAR) marker specific for 3V chromosome based on the sequence of a grain size-related gene *DvGS5* in *D*. *villosum*, an orthologue of *TaGS5* from wheat. The SCAR marker was designated DvGS5-1_443_, which could successfully amplify a unique 3V-specific fragment in CD-3 and *D*. *villosum*, suggesting that this SCAR marker could facilitate targeting the chromosome 3V in the genetic background of wheat for wheat improvement.

## Introduction

Stripe (or yellow) rust, caused by *Puccinia striiformis* Westend. f. sp. *tritici* Erikss. (*Pst*), is considered one of the most dangerous diseases of wheat (*Triticum aestivum* L.) worldwide, and also in China [1]. It was estimated to commonly reduce crop yield by 10–70%, and even up to 100% [2]. The development of disease-resistant wheat cultivars has been suggested to be the most effective, economical, and environmentally friendly strategy to control stripe rust [3,4]. To date, numerous *Yr* (yellow rust) genes have been identified, and they have been officially designated as *Yr1*- *Yr78* [5]. However, concerning the coevolution of plants and pathogens, many of the extensively used *Yr* genes, such as *Yr9* and *Yr26*, did not confer adequate resistance to newly emerging *Pst* strains [6]. Therefore, there is an urgent need for exploring and identifying novel and effective resistance genes against newly emerged *Pst* strains. The progenitors and relatives of crops are immensely valuable for modern agriculture, by providing a wide diversity of desirable genetic resources for plant breeding[7]. In particular, a substantial body of evidence supports that wild relatives of wheat constitute a valuable gene pool for disease resistance in wheat[8–10]. For example, chromosome arm 1RS of rye harboring powdery resistant genes (*Pm8* and *Pm17*) and rust resistance genes (*Sr31*, *Lr26* and *Yr9*) [11–12], and 6VS arm of *Dasypyrum villosum* carrying powdery mildew resistant gene (*Pm21*) [13] are prevalent in wheat commercial cultivars.

*Haynaldia villosa* (L.) Schur (syn. *Dasypyrum villosum* L. Candargy, 2n = 2*x* = 14, VV) is of interest as a genetic germplasm source, possessing many agronomically important traits for wheat improvement, such as tolerance to biotic and abiotic stresses, and high nutritional and bread-making quality [14–15]. Since the development of the first set (#1) of *Triticum*-*Dasypyrum* alien lines [16], the chromosomes of different *D*. *villosum* accessions have been introduced into wheat (set #2 to set #4) [17]. The desirable genes present on V genome chromosomes have been identified and characterized in the genetic background of wheat. For example, the well-known powdery mildew resistant gene *Pm21*, located on 6VS, has been cloned [18–19] and further used in wheat breeding [20]. However, limited progress has been made in the exploration of stripe rust resistant gene (s) in *D*. *villosum* [21].

An effective strategy for utilizing plant genetic resources by employing conventional breeding, molecular genetics, and transformation is gaining ground nowadays [22]. In this study, using functional molecular markers and cytogenetic methods, we characterized a new wheat-*D*. *villosum* 3V (3D) substitution line CD-3 showing high resistance to stripe rust.

## Materials and methods

### Plant materials

*D*. *villosum* accession PI 257477 (genome VV, 2n = 2*x* = 14) was obtained from the National Genetic Resources Program, United States Department of Agriculture. Chinese Spring (CS)—*D*. *villosum* addition lines (# 3) Additions 1V, 2V, and 4V-7V were provided by the School of Life Science and Technology, University of Electronic Science and Technology of China (the *D*. *villosum* accession used to develop this set (#3) of additional line was TA10220). Line CD-3 and the sixty plants of CD-3 used for stripe rust test were the F_6_ progeny derived from hybridization of CS and CS-*D*. *villosum* 3V addition line (# 3). F_2_ population used for genetic analysis of resistance to stripe rust was derived from crosses between CS and CD-3. Other accessions, including common wheat cultivars CS, Chuanmai49, Chuanmai50, Chuanmai60, and rye cultivar JZHM were maintained by our laboratory.

### Non-denaturing fluorescent in situ hybridization (ND-FISH) procedures

Root tips from 60 individual seedlings of CD-3 were collected, treated with nitrous oxide for 2h and fixed with 90% acetic acid for 8–10 min. Then, the root tips were washed quickly with dd H_2_O, and stored in 70% ethanol at -20°C. After being washed with dd H_2_O, the root tips were digested with 1% pectolyase and 2% cellulase solution (Yakult Pharmaceutical Industry Co., Ltd, Tokyo, Japan) as the procedures described by Kato et al. [23]. The oligonucleotides Oligo-pSc119.2, Oligo-pTa-535, Oligo-(GAA)_7_, and Oligo-pHv62-1 used as probes, among which, Oligo-pSc119.2 combined with Oligo-pTa-535 could identify all 42 chromosomes of CS common wheat, as described by Tang et al. (2014), Oligo-(GAA)_7_ could distinguish all chromosomes of B sub-genome as described by [24], and Oligo-pHv62-1 could highlight the 3V chromosome of *D*. *villosum* as reported by Li et al. [25]. The probes mentioned above were synthesized by Invitrogen (Shanghai, China) as described by Tang et al. [26] and Li et al. [25]. ND-FISH analysis was performed as described by Fu et al.[27]. At least three metaphase plates per seedling were analyzed and FISH images were captured using Leica DM2500 microscope (Leica, Shanghai, China).

### PCR-based Landmark Unique Gene (PLUG) marker analysis

Genomic DNA was isolated from young leaves using the CTAB method[28]. PLUG primers were designed as described by Ishikawa et al. [29]. PCR was conducted using a T100^TM^ Thermal cycler (Bio-RAD Laboratories, Emeryville, CA, USA) in a 25 μL reaction mixture, containing 2.5 μL of 10× buffer (50 mM KCl, 1.5 mM MgCl_2_, and 10 mM Tris-HCl, pH 8.3), 200 nmol of each dNTP, 100 ng of genomic DNA, 0.2 U of *Taq* polymerase (TianGen, Beijing, China) and 400 nmol of each primer. The amplification protocol as follows: initial denaturation at 94°C for 3 min; followed by 35 cycles of denaturation at 94°C for 1 min, annealing at 55°C (dependent on different primer sets) for 1 min, extension at 72°C for 2 min, and final extension at 72°C for 10 min. The PCR products were separated on 2% (w/v) agarose gels, and visualized by EtBr staining.

### Stripe rust resistance tests

The sixty plants of CD-3 identified by ND-FISH were grown in the field and used for stripe rust resistance tests. During the cropping seasons in 2014, 2015, 2016 and 2017, field tests were conducted in Pixian city, Sichuan, China. The mixed *Pst* strains, mainly consisting of CYR32, CYR33, and CYR34, were used to infect adult plants of CD-3, CS, and *D*. *villosum*. The infection type (IT) were recorded 18–20 days after inoculation.

Genetic analysis of resistance to stripe rust was conducted on 52 plants of F_2_ (CD-3/CS). After identified by ND-FISH, seeds were grown in small pots(5×5×5cm), and one pot was for one seed. Seedlings at two-leaf stage were inoculated with mixed urediniospores and were kept in dew chamber at 9–12°C for 24 h without light. And then, the seedlings were transferred to rust-free greenhouse with daily cycle of 12 h of light and 12 h of dark at 11°C-17°C. The mixed *Pst* strains mentioned above were used to infect seedling plant. The infection types were recorded 15 days after inoculation.

Infection types were recorded on 1–9 scale as described by Line and Qayoum [30], where IT 0–3 were resistant, IT 4–6 were intermediate, and IT 6–9 were susceptible. The *Pst* strains used were provided by the Plant Protection Institute, Sichuan Academy of Agricultural Sciences, China, and the Plant Protection Institute, Gansu Academy of Agricultural Sciences, China.

### Development of 3V-specific molecular markers

To monitoring the 3V chromosomes from *D*.*villosum*, we chose TaGS5 genes mapped on 3AS and 3DS of wheat for the developing the 3V-specific marker. Five primer pairs for *TaGS5* genes (*Ta*GS5-P5, *Ta*GS5-P6, *Ta*GS5-P7, *Ta*GS5-P8, and *Ta*GS5-P9), were used in this study, as described by Wang et al. [31]. PCR was conducted using a T100^TM^ Thermal cycler (Bio-RAD Laboratories). The reaction system and procedure were in accordance with the description of Wang et al. [31]. The amplified products were separated on 2% (w/v) agarose gels. *D*. *villosum*-specific bands were excised and purified using gel extraction kit (TianGen Biotech) following the manufacturer’s instructions. After introducing the purified product into the vector pMD19-T (TaKaRa Biotechnology, Dalian, China) following the manufacturer’s instructions, the modified vector was transformed into competent cells of *Escherichia coli* strain *DH-5*α. The obtained clones were screened by PCR using M_13_ universal primers, and three positive clones were randomly chosen for double end sequencing at Shanghai Sangon Biotech Co., Ltd, Shanghai, China. Sequences obtained were assembled by DNAman (Lynnon Biosoft, San Ramon, CA, USA).Based on sequence alignment between *DaGS5* and *TaGS5*, putative SCAR primers were designed based on the low-homology region using Primer Premier 5.0 (PREMIER Biosoft, Palo Alto, CA, USA), followed by synthesis at Shanghai Sangon Biotech Co., Ltd. PCR was conducted by a T100^TM^ Thermal cycler (Bio-RAD Laboratories), using a 25 μL reaction system, containing 2.5 μL of 10× buffer (50 mM KCl, 1.5 mM MgCl_2_, 10 mM Tris-HCl, pH 8.3), 40–100 ng of genomic DNA, 200 nmol of each primer, and 1 U of *Taq* DNA polymerase (TianGen Biotech). The PCR protocol was as follows: initial denaturation at 94°C for 5 min, 35 cycles of denaturation at 94°C for 1 min, annealing at 56°C for 0.5 min, extension at 72°C for 0.5 min, and final extension at 72°C for 10 min. The amplicons were separated on a 1% (w/v) agarose gel, and visualized by EtBr staining.

## Results

### Chromosomal characterization

**Fig 1:**
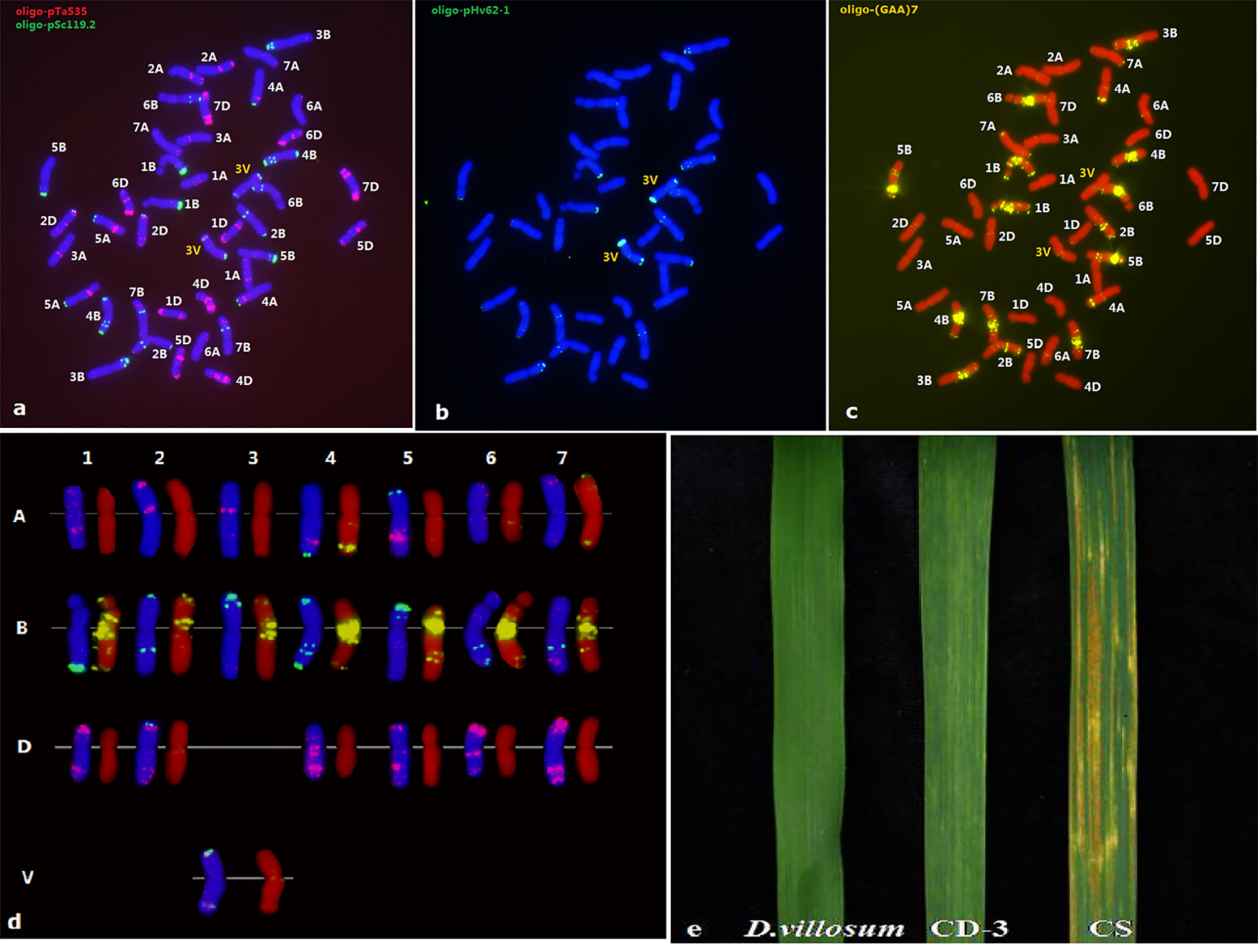
FISH pattern of CD-3 and the leaf response to stripe rust. (a) Chromosomes of CD-3 stained with DAPI (blue), Oligo-pTa535 (red) and Oligo-pSc119.2 (green), (b) chromosomes stained by DAPI (blue) and Oligo-pHv62-1(green), (c) chromosomes stained with propidium iodide, PI (red) and oligo-(GAA)_7_, (d) the FISH karyotype of CD-3, (e) The leaf response to stripe rust of *D*. *villosum*, CD-3 and CS.

Sequential ND-FISH was conducted on the mitotic spread chromosomes of CD-3 using probes of Oligo-pSc119.2, Oligo-pTa535, Oligo-pHv62-1 and Oligo-(GAA)_7_ (Fig 1A–1C). As shown in Fig 1D, we observed that all wheat and *D*. *villosum* chromosomes could be accurately distinguished using the probes mentioned above. The Oligo-pSc119.2 and Oligo-pTa535 probe combination could easily distinguish all 42 wheat chromosomes (Fig 1A). As shown in Fig 1B, Oligo-pHv62-1 highlighted a pair of 3V chromosomes of *D*. *villosum* with strong hybridization signals on the terminal regions of both chromosomal arms, and a faint signal in the centromeric region. Oligo-(GAA)_7_ was mainly located in centromeric or sub-terminal regions of the B genome of wheat (Fig 1C). The chromosome number of CD-3 was 42, containing 40 wheat chromosomes and a pair of 3V chromosomes. By comparing the standard wheat karyotype obtained by the combined use of Oligo-pSc119.2 and Oligo-pTa535 as probes [26], we deduced that wheat chromosome 3D was absent in the line CD-3.

### PLUG marker analysis

**Fig 2:**
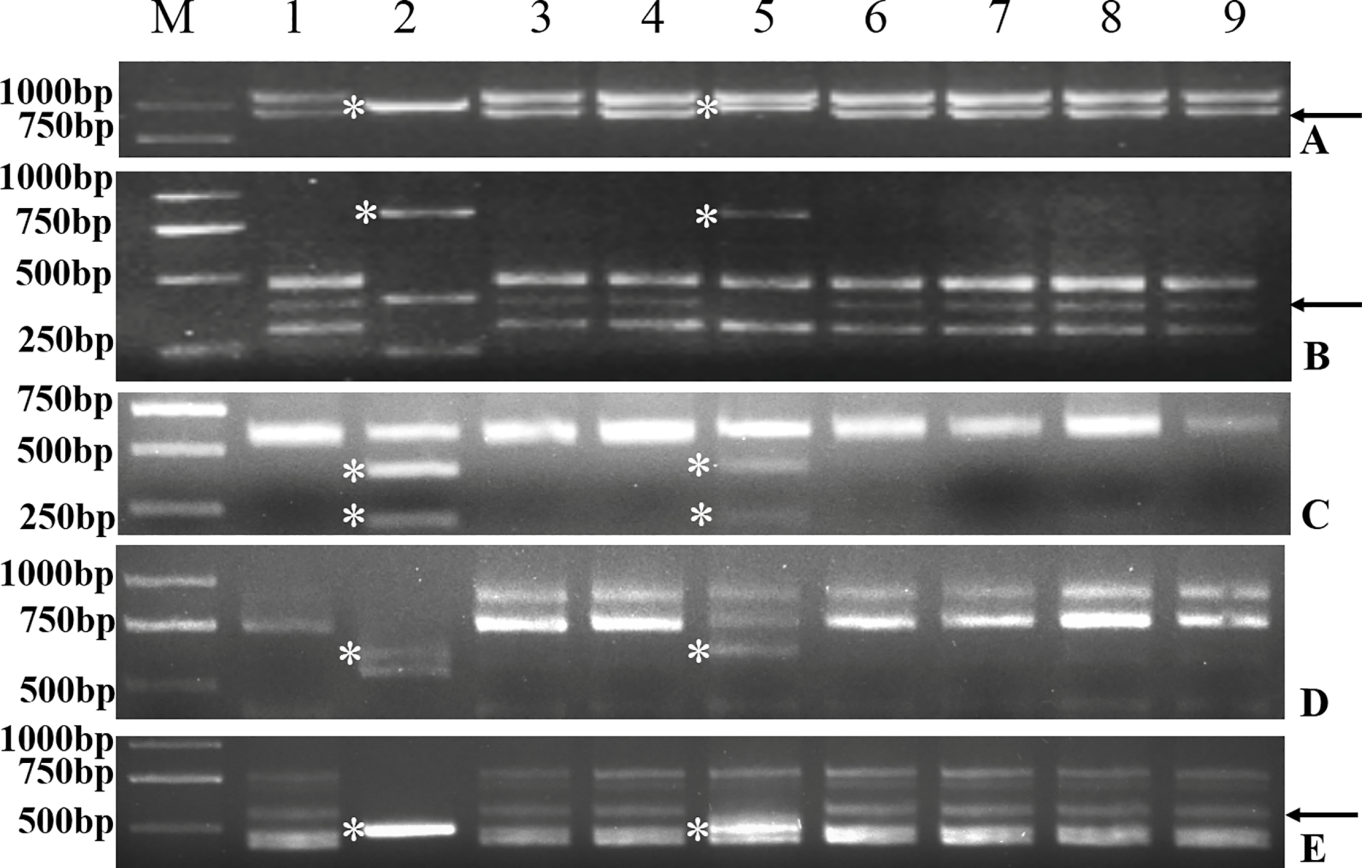
PCR products of PLUG markers in wheat-*D*. *villosum* alien introgression lines. (a)-(e) PCR amplification generated by markers TNAC1248, TNAC1294, TNAC1301, TNAC1277 and TNAC1267, respectively; M, Trans2k plus DNA markers (Trangene, Beijing, China); 1, common wheat CS “Chinese Spring”; 2, *D*. *villosum*, 3–4, CS-*D*. *villosum* 1V -2V additional lines; 5, CD-3; 6–9, CS-*D*. *villosum* 4V -7V additional lines. The asterisks indicated the *D*. *villosum*-specific bands and the arrows showed the 3D-specific band which was absent in CD-3.

*D*. *villosum* was firstly analyzed by employing 30 PLUG markers specific for wheat homoeologous group 3 chromosomes. Five pairs (three were located on the short arm, and two were located on the long arm) generated stable, clear bands in CS, *D*. *villosum*, CD-3, and some CS-*D*. *villosum* addition lines (Table 1). Among the five PLUG primers pairs, three primer pairs (TNAC1248, TNAC1294 and TNAC1267) generated *D*. *villosum*-specific bands in both *D*. *villosum* and CD-3, excluding one fragment from CS, while the remaining two primers (TNAC1301 and TNAC1277) amplified all fragments from *D*. *villosum* and CS (Fig 2). By comparing the CS band pattern with the standard bands obtained in nullisomic-tetrasomic lines of CS using the same primer pairs (TNAC1294 and TNAC 1267) described by Ishikawa et al. (2009), we observed that the fragment absented in CD-3 belonged to chromosome 3D. These results showed that CD-3 contained the *D*. *villosum* 3V chromosome, and had lost a pair of 3D chromosomes.

**Table 1:** The PLUG primers belonging to *Triticeae* homoeologous Group 3 used in the study.

Marker Name	Primer Sequence (5’-3’)	Wheat Bin Map Location	Wheat Chromosomal Location*	Restriction Enzymes	Length of 3V Bands, bp
TNAC1294	F: CGGAAACTTTAGCCTTCTGCT	3AS4-0.45–1.00	3AS-36.23	TaqI	750
	R: GTCGTGTCAGATGCTTTGGAT	3BS9-0.57–0.78	3BS-43.74		
		3DS4-0.59–1.00	3DS-26.26		
TNAC1301	F: TGGTTTCAGATGCAGGAACTT	3AS4-0.45–1.00	3AS-84.19	HaeIII	380/180
	R: CACTAAGGCATGCTGAAGGAG	3BS9-0.57–0.78	3BS-117.93		
		3DS4-0.59–1.00	3DS-71.97		
TNAC1248	F: ATGATGCAGCAGCAAATTACA	3AS4-0.45–1.00	3AS-211.50	-	1000
	R: CTGAGGAGCCTCTCCAACTCT	C-3BS1-0.33	3BS-251.65		
		3DS3-0.24–0.31	3DS-172.90		
TNAC1267	F: GAGAGGCAGCTTCACTAGCAG	3AL3-0.42–0.61	3AL-522.17	-	500
	R: CGTCAGGATCAGCTCTCATGT	3BL2-0.22–0.41	3BL-527.29		
		3DL1-0.23–0.81	3DL-401.87		
TNAC1277	F: AAAGCACCACCACATATGAAA	3AL4-0.61–0.78	3AL-649.02	TaqI	630
	R: GAGGCAGAGAGTGCAAATGTT	3BL7-0.63–0.81	3BL-676.61		
		3DL1-0.23–0.81	3DL-514.27		

Note

*, The information was obtained from website https://urgi.versailles.inra.fr/blast/

### Evaluation of stripe rust resistance

**Fig 3:**
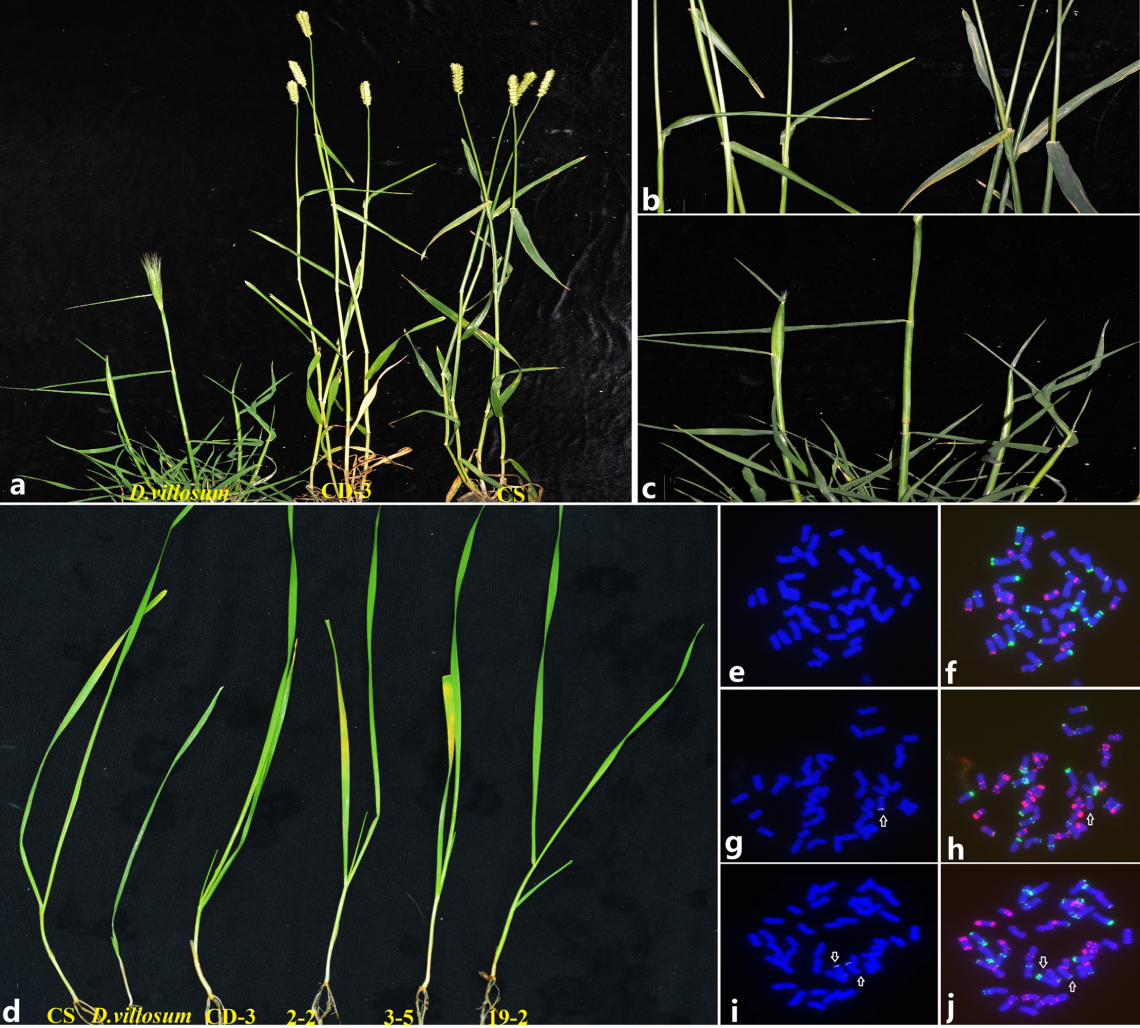
The phenotypic response to stripe rust of *D*. *villosum*, CD-3, CS and 3 plants of F_2_ (CD-3/CS). (a-c) The response to stripe rust of *D*.*villosum*, CD-3 and CS at adult stage. (d) The response to stripe rust of CS, *D*.*villosum*, CD-3 and F_2_ (CD-3/CS) plants at seedling stage. 2–2 was the F_2_ plant without 3V chromosome, 3–5 was the F_2_ plant holding one 3V chromosome, and 19–2 was the F_2_ plant having two 3Vchromosomes. (e-j) The FISH pattern of 2-2(e, f), 3-5(g-h) and 19-2(i-j). Chromosomes were stained by DAPI(blue), Oligo-pHv62-1(white), Oligo-pTa535 (red) and Oligo-pSc119.2 (green). The arrows showed 3V chromosomes.

After field resistance evaluations were conducted in four successive seasons, we found that all 60 individuals of CD-3 and the *D*. *villosum* plants were highly resistant to the epidemic *Pst* strains, including CYR32, CYR33 and CYR34, whereas the recipient parent CS was susceptible (Fig 3A–3C). The resistance tests on 52 plants of F_2_ were conducted at seedling stage. We observed that 7 plants possessing two 3V chromosomes were highly resistant to stripe rust, 10 plants carrying one 3V chromosome and 35 plants without 3V chromosome were susceptible to stripe rust (Fig 3D–3J). These results showed that stripe rust resistance of CD-3 probably originated from recessive gene(s) on the 3V chromosome of *D*. *villosum*.

### Isolation of *GS5* gene from *D*. *villosum* and development of 3V-specific marker

**Fig 4:**
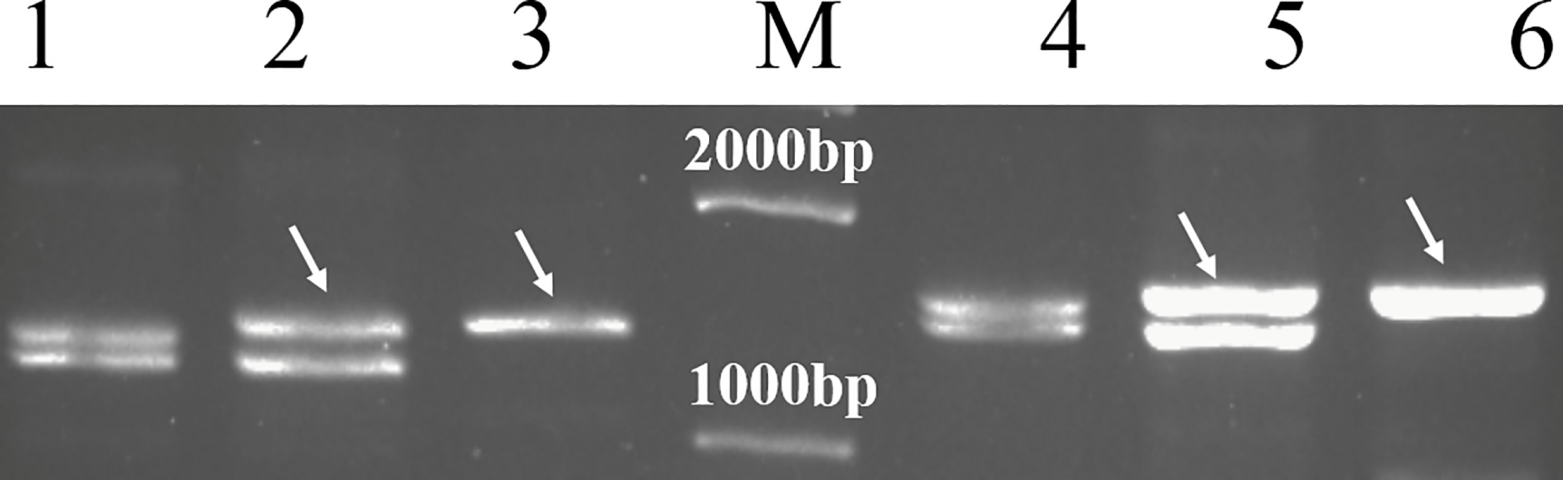
PCR products of primer pairs *Ta*GS5-P6 and *Ta*GS5-P7 in CS, CD-3 and *D*. *villosum*. M, trans2k plus DNA Marker; 1–3, the bands amplified by *Ta*GS5-P6 in CS, CD-3 and *D*. *villosum*, respectively; 4–5, the bands amplified by *Ta*GS5-P7 in CS, CD-3 and *D*. *villosum*. The white arrows showed the *D*. *villosum*-specific bands.

The yield-related gene *TaGS5*, present in 3AS and 3DS of common wheat, was used in this study for developing a 3V-specific SCAR marker. PCR analysis was conducted for CS, *D*. *villosum* and CD-3 using five primer pairs for *TaGS5* genes (*Ta*GS5-P5, *Ta*GS5-P6, *Ta*GS5-P7, *Ta*GS5-P8, and *Ta*GS5-P9) as described by Wang et al. [31] (Table 2). Among them, *Ta*GS5-P6 and *Ta*GS5-P7 could amplify about 1500-bp and 1600-bp fragments (designated DvGS5-P6_1500_ and DvGS5-P7_1600_) from *D*. *villosum*, and CD-3 (Fig 4), respectively, which were slightly different from those obtained from CS. Therefore, the DvGS5-P6_1500_ and DvGS5-P7_1600_ were cloned and sequenced bidirectionally. The obtained DvGS5-P6_1500_ and DvGS5-P7_1600_ were 1380-bp and 1594-bp long, respectively, which could be further assembled into a1829-bp fragment, designated *DvGS5-1*.

**Table 2:** Primers used for the identification of *GS5* gene.

Primer	Primer sequence (5'-3') *	Annealing temperature(°C)	The size (bp) of PCR fragment in CS	The size (bp) of PCR fragment(s) in CD-3	The size (bp) of PCR fragment in *D*. *villosum*
*Ta*GS5-P5	Forward: GCGAACCAAGACAAGCAG Reverse: CCTTGTACTGCGGAAACCTC	56	930	930	930
*Ta*GS5-P6	Forward: CTTCTGAGCTAGGACCTCTC Reverse: ACAAGGTCAGCTAGTTGTGG	56	1226	1226/1380	1380
*Ta*GS5-P7	Forward: ACATCCTCTGACCTCACCAA Reverse: GATACAACTGCATGGCTCCA	57	1427	1427/1594	1594
*Ta*GS5-P8	Forward: TCATTATGTGCCACAACTAGCT Reverse: AGTACCGAAAAGTTGTACGACT	57	1225	1225	1225
*Ta*GS5-P9	Forward: TGTCAATGGGATGTTGCCTG Reverse: TCATCGGTGTGTAGGAAGCTG	58	1162	1162	1162

Note

* Primer sequences were referred to Wang et al. [31]

**Fig 5:**
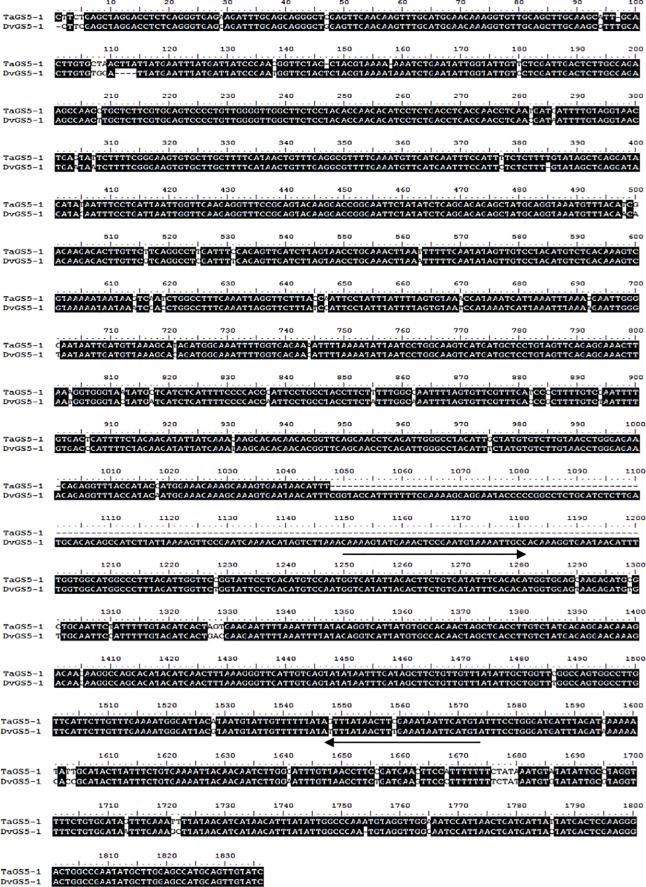
Sequence alignment of DvGS5-1 and TaGS5-1. Arrows showed binding sites of SCAR primer pair DG-3VF and DG-3VR.

**Fig 6:**
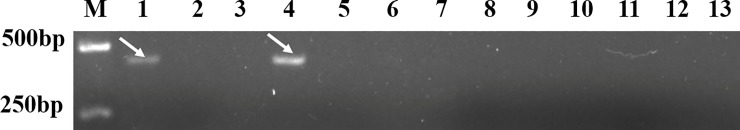
PCR bands generated by DG-3VF and DG-3VR. 1, *D*. *villosum*; 2–3, CS -*D*. *villosum* 1V-2V additional lines; 4, CD-3; 5–8, CS-*D*. *villosum* 4V-7V additional lines; 9–13, common wheat cultivars CS, Chuanmai49, Chuanmai50, Chuanmai60, and rye cultivar JZHM. White arrows indicated the 3V chromosome-specific marker DvGS5-1_443_.

The alignment of *DvGS5-1* and the corresponding region of *TaGS5* (designated *TaGS5-1*) revealed that a 155-bp insert at position 1048–1200, as well as a few short deletions and SNPs, were present in *DvGS5*-1 (Fig 5). Based on this 155-bp fragment insertion, one primer pair, DG-3VF (5’-AGTTCCGAATCAAAACATAGTC-3’) and DG-3VR (5’-AAATCACAATCCTTCTTTATGC-3’) was further designed, and used for analyzing *D*. *villosum*, CD-3, CS-*D*. *villosum* 1V-2V and 4V-7V addition lines, rye JZHM, and several common wheat cultivars, including CS, Chuanmai49, Chuanmai50, and Chuanmai60. A targeted 443-bp band, designated *DvGS5-1*_*443*_, could only be obtained from *D*. *villosum* and CD-3; however, no PCR product was observed in the other materials (Fig 6).

## Discussion

Due to its resistance to several serious wheat diseases, including powdery mildew, rust, eyespot, take-all, and so on [17], *D*. *villosum* has been extensively used as a valuable genetic resource for wheat improvement. In the past few decades, *D*. *villosum* chromatin from several *D*. *villosum* accessions has been introduced into the genetic background of wheat, and several resistant genes have been identified and mapped on individual V-genome chromosomes [17]. For example, chromosome 1V possesses a resistance gene(s) against common bunt (*Tilletia tritici*) [32] and eyespot [33] as well as genes for enhancing wheat quality [15]. Chromosome 2V carries eyespot resistance gene(s) [33] and gene(s) for increasing wheat yield [34]. The 3V chromosome has resistance genes against take-all (*Gaeumannomyces graminis*) and eyespot [33,35], the 4V chromosome carries the eyespot resistance gene *Pch3* [36–37], wheat spindle streak mosaic virus (WSSMV) resistance gene *Wss1* [38]. Chromosome 5V possesses the powdery mildew resistance gene *Pm55*[10] and the 6V chromosome has the powdery mildew resistance gene *Pm21* [13], rust resistance genes *Lr6V#4* [32]and *SrHv6* [39], as well as the CCN resistance gene *CreV* [9].

To date, a total of 78 stripe rust resistance genes in wheat have been officially designated (*Yr1*-*Yr78*). Among them, *Yr30* and *Yr57* have been mapped on chromosome 3B [40–41], *Yr45* and *Yr71* has been placed on 3D [42–43], and *Yr76* has been mapped on 3A[44]. The resistance genes mentioned above, located on the *Triticeae* homoeologous group 3 chromosomes, all originated from hexaploid landraces. No previous studies on stripe rust resistance gene(s) present on chromosome 3V originating from the relative of wheat, *D*. *villosum*, have been reported.

Meanwhile, previous studies proposed that resistance in different *D*. *villosum* accessions may vary. For example, He et al. [45] identified four *Pst*- susceptible *D*. *villosum* accessions from a panel of 110 accessions. Similarly, Yildirim et al. [37] used 115 *D*. *villosum* accessions for analyzing *Pst* resistance, and observed that 33 accessions were resistant to one or more stripe rust fungal strains, and eight accessions were resistant to all strains. These studies implied that different resistance genes exist in different *D*. *villosum* accessions. Thus, it is necessary to continuously screen for novel resistance gene (s) from different *D*. *villosum* accessions and wheat-*D*. *villosum* derived lines. In this study, by using ND-FISH and molecular markers, we identified a novel wheat-*D*. *villosum* 3V (3D) substitution line (CD-3) from the progeny of crosses between CS and the CS-*D*. *villosum* 3V addition line. Moreover, ND-FISH analysis showed a strong Oligo-pSc119.2 signal on the terminal region of 3VL of CD-3, yet not at sub-terminal site of 3VL, suggesting that the *D*. *villosum* chromatin introduced into CD-3 might have originated from a *D*. *villosum* accession different from that used in a previous study by Li et al. [25]. More importantly, the test of *Pst* resistance at adult stage and the genetic analysis of resistance to stripe rust on seedlings of F_2_ population (CD-3/CS) showed that chromosome 3V of CD-3 probably carried high degree of stripe rust resistance which should be controlled by recessive gene(s), and the resistance gene(s) could function in the genetic background of wheat. Therefore, CD-3 should be considered as a valuable resource for further exploration and utilization in wheat breeding.

PCR-based species-specific markers have proven effective tools to monitor alien chromatin harboring valuable genes in the genetic background of wheat [46]. To date, continuing efforts have been made to develop V genome-specific SCAR markers, as well as a few V chromosome-specific SCAR markers [47–50]. However, no studies on the development of 3V-specific SCAR markers have been reported. In this study, we identified a 155-bp insertion into *DvGS5*, an orthologue of *TaGS5*, locating on 3AS and 3DS of common wheat. This helped us in developing a marker to target 3V chromosome in the further transferring of 3V chromosome carrying stripe rust resistance to various wheat genetic background. Based on the polymorphism between *DvGS5* and *TaGS5* we developed a SCAR marker, designated DvGS5-1443, which could generate a 443-bp band specific to 3V chromosome. We demonstrated that it could indicate the presence or absence of the 3V chromosome in the background of wheat reliably, easily and efficiently. Therefore, it could be used as an efficient tool for monitoring the *D*. *villosum* 3V chromosome carrying stripe rust resistant gene (s) for use in wheat breeding programs.

Chromosomal number and structural changes have been monitored and described in numerous wheat-alien genetic stocks, especially the wheat-rye derived lines [51–55]. For wheat-*Dasypyrum* derived lines, Zhang et al. [56] described structural changes on the short arm of chromosome 6D in the CS-*D*. *villosum* nullisomic-tetrasomic (6A/6D) addition (6V) line using Oligo-pTa535 as a probe. Li et al. [25] observed structural changes on chromosomes 1B, 2B, and 7A of a wheat CS-*D*. *breviaristatum* partial amphiploid and chromosomes 1D and 3D of wheat- *D*. *breviaristatum* 7V^b^ addition line. In this study, we detected structural aberrations on chromosome 7B, 4D, and 6D of the wheat-*D*. *villosum* 3V (3D) substitution line CD-3 using Oligo-pSc119.2 and Oligo-pTa535 as probes, comparing with the ND-FISH karyotype of common wheat, CS [26]. These results indicated that chromosomal structural aberrations possibly arose by introduction of *Dasypyrum* chromatin into genetic background of wheat. The mechanism of chromosomal alteration induced by alien chromosomes requires further exploration.
